# Incidence, Mortality, and Risk Factors of COVID-19 in Nursing Homes

**DOI:** 10.3390/epidemiologia3020014

**Published:** 2022-03-28

**Authors:** Alberto Arnedo-Pena, Maria Angeles Romeu-Garcia, Juan Carlos Gascó-Laborda, Noemi Meseguer-Ferrer, Lourdes Safont-Adsuara, Laura Prades-Vila, Matilde Flores-Medina, Viorica Rusen, Maria Dolores Tirado-Balaguer, Susana Sabater-Vidal, Maria Gil-Fortuño, Oscar Pérez-Olaso, Noelia Hernández-Pérez, Rosario Moreno-Muñoz, Juan Bellido-Blasco

**Affiliations:** 1 Epidemiology Division, Public Health Center, 12003 Castelló de la Plana, Spain; romeu_man@gva.es (M.A.R.-G.); gasco_jua@gva.es (J.C.G.-L.); meseguer_noe@gva.es (N.M.-F.); safont_lou@gva.es (L.S.-A.); rusen_vio@gva.es (V.R.); bellido_jua@gva.es (J.B.-B.); 2 Public Health and Epidemiology (CIBERESP), 28029 Madrid, Spain; 3 Department of Health Science, Public University of Navarra, 31006 Pamplona, Spain; 4 Health Programs, Public Health Center, 12003 Castelló de la Plana, Spain; prades_lauvil@gva.es (L.P.-V.); flores_mat@gva.es (M.F.-M.); 5 Microbiology Laboratory, Universitary General Hospital, 12004 Castelló de la Plana, Spain; tirado_dolbal@gva.es (M.D.T.-B.); sabater_sus@gva.es (S.S.-V.); 6 Clinical Analysis and Microbiology Laboratory, Universitary Hospital de la Plana, 12540 Vila-Real, Spain; gil_marfor@gva.es (M.G.-F.); perez_oscola@gva.es (O.P.-O.); hernandez_noeper@gva.es (N.H.-P.); 7 Department of Epidemiology, School of Medicine, Jaume I University, 12006 Castelló de la Plana, Spain; moreno_rosmuny@gva.es

**Keywords:** COVID-19, nursing homes, incidence, mortality, risk factors, epidemiological surveillance, ecological design

## Abstract

During the period from March 2020 to January 2021, we performed an analysis of incidence, mortality, and risk factors of COVID-19 in nursing homes (NHs) in two health departments (HDs) of Castellon (Spain) 2021 through epidemiological surveillance and an ecological design. Laboratory-confirmed COVID-19 cases, cumulative incidence rate (CIR), and mortality rate (MR) of 27 NHs were collected. Information of residents, staff, and facilities was obtained by questionnaire. Multilevel Poisson regression models were applied. All NHs in the HDs participated with 2229 residents (median: 83 years old, 67.3% women) and 1666 staff. Among residents, 815 cases (CIR: 34.8 per 100) and 202 deaths (MR: 8.7 per 100, case fatality 21.0%) were reported and, among staff, 296 cases (CIR: 19.2 per 100) without deaths. Residents’ CIR and MR increased with staff CIR, age of the building, residents/staff ratios, occupancy rate, and crowding index; CIR increased with private NH ownership, large NH size, large urban area, and the percentage of women residents; and MR was associated with residents’ severe disabilities. In conclusion, several risk factors of COVID-19 incidence and mortality can be prevented by improving infection and quality controls, ameliorating residents/staff ratios, improving structural facilities, and increasing NH public ownership to avoid new outbreaks.

## 1. Introduction

Nursing homes (NHs) have suffered the most devastating effects of COVID-19 in terms of incidence and mortality in many countries [1,2,3], including Spain [4,5,6], where it has been reported that deaths from COVID-19 in NHs reached 66% of total deaths during the first wave of the pandemic [7]. NH residents have an elevated risk of moderate to severe COVID-19 considering advancing age, deficient immune response, and the prevalence of chronic diseases. In addition, hygienic conditions, environmental structures, and staffing of NHs have been found to be key players in COVID-19 transmission [8,9]. Control and prevention of COVID-19 require studies on the epidemiological situation and the potential factors that influence disease outcomes [10,11]. Frequently, COVID-19 studies in NHs have been focused on singular outbreaks; however, long period epidemiological surveillance across a territory is less frequent [12,13,14,15].

The NHs of Health Departments 2 and 3 of Castellon (Valencia Community Spain) suffered a high incidence and mortality from COVID-19 until the start of anti-SARS-CoV-2 vaccination in January 2021, but differences among the NHs were reported during the period. The analysis of such differences can be potentially useful to help prevent new COVID-19 outbreaks.

The aim of the study was to estimate the incidence and mortality of COVID-19 in NH residents, and their associated risk factors, before starting the vaccination against the disease.

## 2. Materials and Methods

### 2.1. Explanation

The study consisted of two parts. First, during the period from March 2020 to January 2021, epidemiological surveillance of the incidence and mortality of COVID-19 in the NHs of Health Departments 2 and 3 of Castellon (Valencia Community, Spain) was implemented. Second, an ecological design to estimate potential risk factors associated with the transmission of COVID-19 was performed with the aggregate data of residents, staff, and facilities. During the study period, the population of the two health departments was 464,398 inhabitants, and the population age of 65 years old and above represented 18.7%.

The study was performed by the Epidemiology Division of the Public Health Center of Castellon and the Microbiology’s Services of the University General Hospital of Castellon and University Hospital of La Plana in Vila-real. To carry out the study, data of cases and deaths of COVID-19 were obtained from the reports of NH outbreaks in all NHs of the two health departments, both for residents and staff. In the control and prevention of COVID-19 transmission in the NHs, several measures were implemented, including the screening of all residents and staff of the NHs to detect SARS-CoV-2 infections, except in one NH with 39 residents at the beginning of the COVID-19 epidemic.

During the study period, 100% of COVID-19 cases and deaths were confirmed by RNA detection of SARS-CoV-2 by the real-time reverse transcriptase-polymerase chain reaction (RT-PCR) test, using Roche Lightmix Modular SARS-CoV-2 (Roche-TIB MOLBIOL D-12103 Berlin, Germany) [16], VIASURE SARS-CoV-2 Real Time PCR Detection Kit (CerTest Biotec S.L. San Mateo de Gallego, Zaragoza, Spain), Abbott Real-Time SARS-CoV-2 (Abbott Laboratory, Abbot Park, IL, USA), and Argene SARS-CoV-2 R-Gene (Biomérieux SA, F-69280 Marcy L’Etoile, France). In addition, serological tests, based on chemiluminescence (IgM-S, IgG-S, of IgG-N, Abbott Laboratory, Abbot Park, IL, USA) [17], and antigen detecting rapid diagnostic tests [18] were used in screening studies with RT-PCR confirmation of positive cases. These determinations were performed at the Microbiology Service of University General Hospital of Castellon and, to a lesser extent, at the Clinical and Microbiology Service of University Hospital of La Plana in Vila-real.

To obtain information about NH characteristics, a questionnaire implemented by the health workers of the Public Health Center of Castellon was answered by the direction of each NH. In this questionnaire, the variables included were the following: numbers of residents, age mean, sex distribution, degree of disability, number of staff and their occupation, the age of the building, number of beds, singles and double bedrooms, bedrooms with three beds, number of bathrooms, and a total of bathrooms. In addition, the capacity to compartmentalize within zones, COVID-19 patients’ isolation, and quarantines in the NHs were included in the questionnaire. To complete the study, contingency plans prepared by the NHs for the prevention of the COVID-19 pandemic were consulted. Residents′ hospitalization due to COVID-19 was included in this study, considering the conditions to be hospitalized and the diagnosis required during the pandemic, as well as the medicalization of some NHs, which could bias its validity.

### 2.2. Statistical Analysis

In the description of the variables, the median and its ranges and the mean and the standard deviation were used. The cumulative incidence rates (CIR) of COVID-19 in residents and staff were calculated considering the total number of reported cases divided by residents or staff who were residing or working in each NH when the first case of COVID-19 was reported. The mortality rate (MR) of COVID-19 was calculated by dividing the total number of deaths by the total number of residents who were residing at the NHs in the first outbreak. The case fatality was calculated by dividing the number of deaths from COVID-19 by the number of patients from COVID-19. It has been considered that the new entries to the NHs, especially of residents, during the study period were very limited due to the pandemic situation. However, CIR and MR in some NHs could have an increase due to maintaining the initial populations. In the analysis of potential risk factors, one NH was excluded, as it was undergoing renovation with a few residents living there.

Cumulative incidence and mortality rates were the dependent variables, and the independent variables were the following:

− Residents: mean age (years), women percentage (%), degree three of disability (%). The used index of disability, based on level of dependence, comprehended four degrees: 0 (no disability); 1 minor disability (mild dependence); 2 moderate disabilities (maintains autonomy daily life); 3 severe/profound disabilities. Degree three of disability needs help for functions like grooming, dressing, feeding, or going to bed.
− Nursing home: ownership (private or public), size (number of residents less than 100, or ≥100), municipality where NHs were located, large urban area (≥50,000 inhabitants), urban area (≥10,000–49,999 inhabitants), semi-urban area (≥2500–9999 inhabitants), and rural area (≤2500 inhabitants). NHs facilities: number of beds, baths, and bathrooms; in addition, number of single and double bedrooms, and bedrooms with three beds.
− Occupancy rate: resident population/NH beds.
− Staff: number of total staff, number of registered nurses, number of nursing assistants. Ratios: residents/staff, residents/registered nurses, residents/nursing assistant, beds/staff, beds/nurse assistant, and bathrooms/residents.

According to Brown and co-authors, who described a crowding index in a study of COVID-19 risk factors in NHs in Canada [19], we estimated a crowding index with some variation in our specific design by the formula:

Crowding index = number of residents in the first COVID-19 outbreak/[(number of bedrooms/2) + (number of bathrooms/2)].

The crowding index is a mean of singles, doubles, and three beds in bedrooms and bathrooms in relation to the number of residents for each NH. If the index is equal to or larger than 2.00, it could be an indication of crowding.

In each municipality where each NH was located, the COVID-19 cumulative incidence rate per 100,000 inhabitants was collected for the 2020 year.

We used Poisson regression models for the univariate analysis and multilevel Poisson regression models for the multivariate analysis, considering the location area of the reference level of the NHs. Calculation of crude and adjusted relative risks (RRs) with a 95% confidence interval (CI) as a measure of the associations between risk factors and the cumulative incidence and mortality rates was performed. The direct acyclic graph (DAG) approach with DAGitty version 3.0 (Johannes Textor, Nijmegen, The Netherlands) [20] was used to consider potential confounding factors in the multivariate analysis. Each independent variable was adjusted for the confounding factors following the DAGs. All statistical analysis was performed with Stata^®^ version 14.2 (Stata Corp, College Station, TX, USA).

No Ethical Committee approval was necessary considering the epidemiological surveillance of the NHs motivated by the COVID-19 pandemic, the anonymity of all the participants, and the administrative information about the NHs.

## 3. Results

A total of 27 NHs were included in the study, representing 100% of public and private NHs in Health Departments 2 and 3 of Castellon. The study period was from March 2020 to January 2021, before the vaccination anti-SARS-CoV-2 was implemented.

The NHs were located in the 15 following municipalities (inhabitants): eight in Castelló de la Plana (174,262), the administrative capital; three in Vila-real (51,130); two in Almassora (26,878), Benicassim (18,364), and Borriana (35,052); and one in Onda (24,939), Nules (13,256), La Vall D’Uixo (31,549), Torreblanca (5606), Cabanes (3027), Moncofa (6950), L’Alcora (10,428), Vila-Franca (2197), Montan (368), and Villahermosa del Rio (484).

A total of 2229 residents lived in the NHs during the study period, with a workforce of 1666 people, including 104 registered nurses (8.4%) and 733 nursing assistants (44.0%). The ownership was private in 18 NHs and public in 9 NHs. The size of the NHs presented differences ranging from less than 50 residents (2 NHs), from 50 to 99 residents (16 NHs), and 100 residents and above (9 NHs). The mean age of the NH buildings was 19.5 ± 10.0 years (median: 16 years, range: 5–41). The NHs had a total of 2490 beds and 1424 bathrooms with 662 single bedrooms (26.6%), 899 double bedrooms (72.2%), and 10 bedrooms with 3 beds (1.2%). The occupancy rate had a median of 92.9% (range: 52–100%). The characteristics of the residents, staff, facilities, residents/staff ratios, and the current situation regarding the capacity for compartmentalization, isolation, and quarantine are shown in Table 1.

Considerable differences in relation to residents, staff, and facilities were found. The residents’ median age was 83 years old, with a higher percentage of women (67.3%), and the degrees of disability ranged from none (4.3%) to severe/profound (36.3%). The crowding index was 1.64, but with a notable range from 0.70 to 2.13. Regarding compartmentalization, 18.5% of NHs could not carry it out. All NHs had possibilities of isolation and quarantine except one.

During the study period, 46 outbreaks of COVID-19 were reported in the NHs, considering the existence of a single case, either resident or staff, that required special action to control and prevent the spread of the disease. Only a single NHs had no outbreak reported; 14 NHs had a single outbreak, 6 NHs had two outbreaks, 5 NHs had 3 outbreaks, and 1 NH had 5 outbreaks. The median of the number of resident cases per outbreak was 19 (range: 0–115), and the median of the number of staff cases per outbreak was 9 (range: 0–45). The median of deaths in residents per outbreak was 4 (range: 0–35). In the staff, deaths were not reported.

COVID-19 incidence and mortality in the NHs are shown in Table 2. The median of the cumulative incidence rate was 25.3% (range: 0–103.0) in residents and 11.7% (range: 0–63.6%) in staff. Three outbreaks took place in one NH, and the median cumulative incidence rate, considering the initial resident population attained, was 103%. For comparison, the mean cumulative incidence rate was 34.8 ± 35.7 per 100 residents and 19.2 ± 18.2 per 100 staff. Considering residents’ mortality, there were 202 deaths with mean and median mortality rates of 8.7 and 6.3 per 100 residents, respectively. The median of case fatality was 21.0% (range: 0–100%), and the mean was 22.7 ± 22.7.

Crude and adjusted RRs of risk factors for cumulative incidence rates of COVID-19 in the NHs are shown in Table 3 and Table 4. Considering the adjusted analysis (Table 3), the incidence rate was significantly increased with the private ownership NHs (aRR = 1.45 95% CI: 1.21–1.74), size of 100 residents or more (aRR = 1.39 95% CI: 1.10–1.77), NHs located in large urban areas (aRR = 2.14 95% CI: 1.28–3.56), and higher percentage of women residents (aRR = 3.1 95% 1.11–8.53). Severe disability and age were not associated with cumulative incidence rates of residents.

In the adjusted analysis (Table 4), the cumulative incidence rate of residents significantly increased with a higher incidence rate of the staff (aRR = 49.7 95% CI: 33.0–78.2), age of NH building (aRR = 1.06 95% CI: 1.04–1.08), elevated occupancy rate (aRR = 2.09 95% CI: 1.69–2.58), higher residents/staff ratio (aRR = 1.41 95% CI: 1.09–1.82), higher residents/nursing assistant ratio (aRR = 1.38 95% CI: 1.24–1.54), higher beds/nursing assistant ratio (aRR = 1.11 95% 1.01–1.22), and raised crowding index (aRR = 2.29 95% CI: 1.69–3.10). The incidence rate of municipalities, bathrooms/resident ratio, and beds/staff ratio were not associated with the incidence rate of residents.

Risk factors of the mortality rates are shown in Table 5 and Table 6. Private NHs, size of 100 or more residents, and NHs located in large urban areas had higher mortality rates, but there were no significant associations. In contrast, severe disability was directly associated with a higher mortality rate (aRR = 1.01 95% CI: 1.00–1.02). The mortality rate was significantly increased with a higher incidence rate in the staff (59.90 95% CI: 26.64–134.89), age of the building (aRR = 1.08 95% CI: 1.04–1.11), elevated occupancy rate (aRR = 1.86 95% CI: 1.22–2.82), higher residents/nursing assistant ratio (aRR = 1.44 95% CI: 1.14–1.82), and raised crowding index (aRR = 3.11 95% 1.55–6.26) (Table 6).

## 4. Discussion

The results indicate that incidence and mortality of COVID-19 in the NH residents were associated with staff incidence rate, staffing ratios, and facility characteristics as highlighted risk factors. Notable differences in the outcomes, as well as risk factors between NHs, were observed. However, the medians of the different staffing ratios were among the recommended ratios of the NHs by the Spanish Society of Geriatrics and Gerontology [21].

The risk factors associated with incidence and mortality rates in residents were incidence rate of staff, age of the building, occupancy rate, crowding index, and residents/nurse assistant ratio. Risk factors associated with incidence rate only were high size, large urban area, private ownership, residents/staff ratio, beds/nursing assistant ratio, and percentage of women residents. Residents’ severe disabilities were only associated with mortality. Some of these risk factors have been described in NH studies [22,23,24]. In our study, the incidence rate of COVID-19 in the municipalities was not associated with the NH incidence rate in contrast with other studies [25,26]; this result may be related to small differences in incidence rates among the municipalities and the adoption of preventive measures in the NHs when an increase in COVID-19 incidence in the municipalities was reported. Some studies in NHs did not detect structural facility risk factors in COVID-19 outbreaks during the first wave of the pandemic [27,28]. However, when the period before the anti-SARS-CoV-2 vaccine was considered, several facility characteristics, such as private ownership, large size, high occupancy rates, and high residents/staff ratios, were found associated with incidence and mortality of COVID-19 [29,30].

When comparing our incidence and mortality rates with other research, the study period must be considered, as most of the publications refer to the first wave of the pandemic. In Spain, residents’ incidence rates were estimated to vary from 26.0 to 44.7 per 100 residents and mortality from 8.1 to 11.0 per 100 residents [28,31,32,33], comparable with our results but higher than other developed countries [19,34,35]. A hypothesis to explain the COVID-19 high mortality in the Spanish NHs is the insufficient resources that cause low staff and functional deficiencies; thus, regional NHs deaths were associated with large sizes, high occupancy rate, and high resident places/staff ratio [36]. This is consistent with the information about low salaries and temporary hiring of NH staff [37].

Our results are in line with studies of COVID-19 incidence and mortality in NHs, including crowding [19,38], incidence rates in staff [39,40], private ownership [14,41], high occupancy rate [15,36], high residents/staff ratios [42,43,44], large size [45,46,47], severe disability [48,49], urban areas [47], and aging facility building [10,50], which could be a proxy of the NHs physical environment and equipment, and the older could have more deficiencies than the new. In contrast to our results, some studies have found a higher mortality rate in men than women [48].

Some risk factors found in our study can be prevented by carrying out different measures. First, the improve infection control in residents and staff. Second, reduce occupancy rate and improve residents/staff and residents/nursing assistant ratios. Third, increase single bedrooms with baths. Fourth, increase NH public ownership. Fifth, reduce NH size to obtain compartmentalization zones and isolation for each NH [51,52,53]. Finally, establish surveillance of quality care in NHs [54].

Vaccination against SARS-CoV-2 has made a crucial inflection point in the COVID-19 pandemic [55]. However, it is convenient to keep control and prevention measures in NHs, considering the fragility of residents, the generation of COVID-19 variants with variable transmission capacities, and residents/staff ratios together and NH environmental conditions [56,57].

This study has limitations, including the usage of aggregated data, the retrospective design, and the employment of fixed populations of residents and staff, which could overestimate incidence and mortality rates in some NHs. In addition, a few potential confusion factors of residents and staff were included in the analysis. Further studies could analyze nursing hour distributions in NHs, which have been associated with incidence and mortality rates [58,59,60], as well as variations in occupancy rates [61]. Considering the retrospective design, no hygiene measures carried out to control of COVID-19 pandemic were examined in our study [62,63].

New research will be important to define with more precision the risk factors of COVID-19 transmission in NHs based on the characteristics of residents and staff, facility structures, and external factors [22,64].

## 5. Conclusions

The elevated incidence and mortality rates in the residents were associated with several risk factors which can be prevented by improving infections and quality controls, ameliorating residents/staff ratios, promoting NHs of low size with better facilities, and increasing NH public ownership, in order to avoid new outbreaks.

## Figures and Tables

**Table 1:** Characteristics of 27 nursing homes (NHs). Health Departments 2 and 3 of Castellon. Residents, staff, and NHs facilities. March 2020–January 2021.

Variables	
Residents	N = 2229
Age median of means (range)	83 years (59–87)
Women percentage (median range)	67.3% (33.6–100%)
Disability (median range) ^1^	
0 None	4.3% (0–23.3%)
1Mild/Minor	12.5% (0–66.3%)
2 Moderate	41.6% (16.1–71.55%)
3 Severe/Profound	36.3% (4.0–73.4%)
Staff	N = 1666
Registered nurses	N = 104
Nursing assistants	N = 733
Nursing homes	N = 27
Age of the building (median range) ^2^	16 (5–41) years
Beds	N = 2490
Occupancy rate (median range)	92.9% (52–100%)
Bathrooms	N = 1424
Single bedrooms	N = 662 (41.6%)
Double bedrooms	N = 899 (56.5%)
Three bedrooms	N = 30 (1.9%)
Ratios (median range)	
Residents/staff	1.49 (0.86–2.57)
Residents/registered nurse	23.7 (1.7–130.0)
Residents/nursing assistant	3.23 (0.56–5.53)
Beds/staff	1.56 (0.91–3.09)
Beds/nursing assistant	3.49 (1.84–6.48)
Bathrooms/resident	0.64 (0.1–1.41)
Crowding index	1.64 (0.70–2.13)
Nursing homes’ capacity	
Compartmentalization	22 (81.5%)
Isolation	26 (96.7%)
Quarantines	26 (96.3%)

^1^ Missing information in 1 NH; ^2^ Missing information in 1 NH.

**Table 2:** Incidence and mortality of COVID-19 in nursing homes of Health Departments 2 and 3 of Castellon. March 2020–January 2021.

Total Population	Outcomes	Mean ± SD ^1^	Median (Range)
	Cases ^2^	Cumulative incidence rate	Cumulative incidence rate
Residents	815	34.8% ± 35.7	25.3% (0–103.0)
Staff	296	19.2% ± 18.2	11.7% (0–63.6)
	Deaths	Mortality rate	Mortality rate
Residents	202	8.7% ± 9.4	6.3% (0–30.2)
Staff	0	0	0

^1^ SD = standard deviation. ^2^ In a NH, only the deaths of residents were reported.

**Table 3:** Risk factors of the cumulative incidence rate (CIR) of COVID-19 in residents of nursing homes. Health Departments 2 and 3 of Castellon. March 2020–January 2021.

Variables	CIR ^1^	cRR ^2,3^ 95% CI ^4^	*p*-Value	Arr ^5^ 95% CI	*p*-Value
Ownership					
Private ^6^	25.4%	1.26 (1.08–1.47)	0.003	1.45 (1.21–1.74)	0.000
Public	15.0%	1.00		1.00	
Size ^7^					
N ≥ 100 residents	28.2%	1.04 (0.88–1.24)	0.000	1.39 (1.10–1.77)	0.006
N ≤ 100	7.1%	1.00		1.00	
Large urban area ^8^					
≥50,000 inhabitants.	50.0%	2.46 (2.10–2.86)	0.000	2.14 (1.28–3.56)	0.004
<50,000 inhabitants.	5.6%	1.00		1.00	
Residents (median values)					
Severe disability ^9,10^ > 36.3%	28.2%	1.01 (1.00–1.02)	0.000	1.00 (0.99–1.01)	0.497
≤36.3%	3.3%	1.00		1.00	
Age (mean) ^10,11^ > 82.5 years	15.4%	0.98 (0.97–0.99)	0.000	0.99 (0.98–1.01)	0.364
≤82.5 years	25.4%	1.00		1.00	
Women% ^10,12^ > 67.3%	25.3%	1.13 (0.60–2.13)	0.705	3.01 (1.11–8.53)	0.031
≤67.3%	24.4%	1.00		1.00	

^1^ CIR = cumulative incidence rate (median). ^2^ RR = relative risk. ^3^ c = crude ^4^ CI = confidence interval. ^5^ Adjusted. ^6^ Adjusted for mean age % women disability. ^7^ Adjusted for mean age % women disability ownership. ^8^ Adjusted mean age % women. ^9^ Adjusted for mean age %women. ^10^ Median value of the variable. ^11^ Adjusted for % women. ^12^ Adjusted for mean age.

**Table 4:** Risk factors of the cumulative incidence rate (CIR) of COVID-19 in residents of nursing homes (II). Health Departments 2 and 3 of Castellon. March 2020–January 2021.

Variables (Median Values)	CIR ^1^	cRR ^2,3^ 95% CI ^4^	*p*-Value	aRR ^5^ 95% CI	*p*-Value
CIR municipalities ^6,7^ > 2820 per 100.000	3.7%	1.01 (1.00–1.02)	0.000	1.01 (0.99–1.02)	0.341
≤2820 per 100.000	26.8%	1.00		1.00	
CIR staff ^6,8^ > 11.7 per 100	73.5%	38.37 (28.21–53.40)	0.000	49.7 (33.0–78.2)	0.000
≤11.7 per 100	2.1%	1.00		1.00	
Age of NHs building ^6,9^ > 16 years	7.1%	1.03 (1.02–1.04)	0.000	1.06 (1.04–1.08)	0.000
≤16 years	27.5%	1.00		1.00	
Occupancy rate ^6,9^ > 92.9%	26.1%	1.47 (1.28–1.70)	0.000	2.09 (1.69–2.58)	0.000
≤92.9%	15.4%	1.00		1.00	
Residents/staff ratio ^6,9^ > 1.49	52.0%	1.61 (1.40–1.87)	0.000	1.41 (1.09–1.82)	0.010
≤1.49	15.0%	1.00		1.00	
Residents/nursing assistant ratio ^6,9^ > 3.23	52.0%	1.34 (1.26–1.43)	0.000	1.38 (1.24–1.54)	0.000
≤3.23	15.8%	1.00		1.00	
Beds/staff ratio ^6,9^ > 1.56	28.2%	1.18 (1.05–1.33)	0.000	0.82 (0.65–1.05)	0.111
≤1.56	6.3%	1.00		1.00	
Beds/nursing assistant ratio ^6,9^ > 3.49	25.4%	1.14 (1.09–1.21)	0.000	1.11 (1.01–1.22)	0.024
≤3.49	15.8%				
Bathrooms/resident ratio ^6,9^ > 0.61	5.6%	0.61 (0.44–0.84)	0.000	1.38 (0.84–2.29)	0.205
≤0.61	26.1%	1.00		1.00	
Crowding index ^6,9^ > 1.64	39.4%	1.48 (1.17–1.88)	0.009	2.29 (1.69–3.10)	0.000
≤1.64	5.6%	1.00		1.00	

^1^ CIR = cumulative incidence rate (median). ^2^ RR = relative risk. ^3^ c = crude ^4^ CI = confidence interval. ^5^ Adjusted. ^6^ Median of the variable. ^7^ Adjusted for mean age % women disability NH size ownership. ^8^ Adjusted for mean age % women disability. ^9^ Adjusted mean age % women disability ownership NHs size.

**Table 5:** Risk factors of the mortality rate (MR) of COVID-19 in residents of nursing homes (I). Health Departments 2 and 3 of Castellon. March 2020–January 2021.

Variables	MR ^1^	cRR ^2,3^ 95% CI ^4^	*p*-Value	aRR ^5^ 95% CI	*p*-Value
Ownership					
Private ^6^	7.3%	0.97 (0.72–1.30)	0.825	0.98 (0.69–1.39)	0.902
Public	2.3%	1.00		1.00	
Size ^7^					
N ≥ 100 residents	7.5%	1.47 (1.11–1.95)	0.008	1.04 (0.64–1.70)	0.877
N < 100 residents	1.9%	1.00		1.00	
Large urban area ^8^					
≥50,000 inhabitants	7.7	1.99 (1.48–2.67)	0.000	1.92 (0.60–6.20)	0.273
<50,000 inhabitants	1.6	1.00		1.00	
Next hospital distance (Km)		1.00 (0.99–1.02)	0.677	0.99 (0.97–1.02)	0.596
Residents (median values)					
Severe disability ^9,10^					
>36.3%	7.5%	1.02 (1.01–1.03)	0.000	1.01 (1.00–1.02)	0.045
≤36.3%	1.9%	1.00		1.00	
Age (mean) ^9,11^					
>82.5 years	3.8%	0.99 (0.96–1.01)	0.300	1.00 (0.97–1.03)	0.920
≤82.5 years	6.3%	1.00		1.00	
Women % ^9,12^ > 67.3%	5.0%	0.83 (0.24–2.86)	0.763	4.41 (0.75–25.85)	0.100
≤67.3%	4.9%	1.00		1.00	

^1^ MR = mortality rate (median). ^2^ RR = relative risk. ^3^ c = crude ^4^ CI = Confidence interval. ^5^ Adjusted. ^6^ Adjusted for mean age % women disability. ^7^ Adjusted for mean age % women disability ownership. ^8^ Adjusted mean age % women. ^9^ Median values of the variable. ^10^ Adjusted for mean age % women. ^11^ Adjusted for % women. ^12^ Adjusted for mean age.

**Table 6:** Risk factors of the mortality rate (MR) of COVID-19 in residents of nursing homes (II). Health Departments 2 and 3 of Castellon. March 2020–January 2021.

Variables (Median Values)	MR ^1^	cRR ^2,3^ 95% CI ^4^	*p*-Value	aRR ^5^ 95% CI	*p*-Value
CIR staff ^6,7,8^ > 11.7 per 100	19.5%	45.6 (22.6–96.7)	0.000	59.9 (26.64–134.81)	0.000
≤11.7 per 100	1.3%	1.00		1.00	
CIR municipalities ^6,10^ > 2820 per 100.000	0.9%	1.01 (0.99–1.03)	0.413	1.02 (0.99–1.05)	0.261
≤2820 per 100.000	7.7%	1.00		1.00	
Age of NHs building ^6,11^ > 16 years	6.3%	1.04 (1.02–1.07)	0.006	1.08 (1.04–1.11)	0.000
≤16 years	3.8%	1.00		1.00	
Occupancy rate ^6,9^ < 92.9%	5.0%	1.82 (1.36–2.44)	0.000	1.86 (1.22–2.82)	0.000
≤92.9%	1.5%	1.00		1.00	
Residents/staff ratio ^6,11^ > 1.49	8.7%	0.89 (0.64–1.23)	0.480	0.95 (0.54–1.66)	0.862
≤1.49	2.3%	1.00		1.00	
Residents/nursing assistant ratio ^6,11^ > 3.23	8.0%	1.12 (0.96–1.30)	0.142	1.44 (1.14–1.82)	0.002
≤3.23	2.8%	1.00		1.00	
Bed/staff ratio ^6,11^ > 1.56	7.5%	0.75 (0.58–0.93)	0.033	0.71 (0.45–1.12)	0.144
≤1.56	1.9%	1.00		1.00	
Beds/nursing assistant ratio ^6,11^ > 3.36	7.5%	1.04 (0.99–1.11)	0.095	1.17 (0.96–1.42)	0.120
≤3.36	2.8%	1.00		1.00	
Bathrooms/resident ratio ^6,11^ >0.61	1.9%	0.28(0.13–0.57)	0.000	1.15 (0.38–3.54)	0.801
≤0.61	7.0%	1.00		1.00	
Crowding index ^6,11^ > 1.64	9.5%	2.57 (1.58–4.19)	0.000	3.11 (1.55–6.26)	0.000
≤1.64	1.9%	1.00		1.00	

^1^ MR = mortality rate (median). ^2^ RR = relative risk. ^3^ c = crude ^4^ CI = Confidence interval. ^5^ Adjusted. ^6^ Median value of the variable. ^7^ Cumulative incidence rate (median). ^8^ Adjusted for mean age % women disability. ^9^ Adjusted for mean age % women disability. ^10^ Adjusted for mean age % women disability NH size ownership. ^11^ Adjusted mean age % women disability NH size ownership.

## Data Availability

Authorization of the Public Health Center’s direction will be required to consult the data set of this study.
